# Contemporary review on pediatric hypertrophic cardiomyopathy: insights into detection and management

**DOI:** 10.3389/fcvm.2023.1277041

**Published:** 2024-01-04

**Authors:** Areez Shafqat, Abdullah Shaik, Snygdha Koritala, Ali Mushtaq, Belal Nedal Sabbah, Ahmed Nahid Elshaer, Omar Baqal

**Affiliations:** ^1^ College of Medicine, Alfaisal University, Riyadh, Saudi Arabia; ^2^ Department of Internal Medicine, Ascension St. John Hospital, Detroit, MI, United States; ^3^ Dr. Pinnamaneni Siddhartha Institute of Medical Sciences & Research Foundation, Gannavaram, India; ^4^ Department of Internal Medicine, Cleveland Clinic Foundation, Cleveland, OH, United States; ^5^ Department of Internal Medicine, Creighton University School of Medicine, Omaha, NE, United States; ^6^ Department of Internal Medicine, Mayo Clinic, Phoenix, AZ, United States

**Keywords:** hypertrophic cardiomyopathy, cardiac MRI, sudden cardiac death, myocardial fibrosis, exercise, late gadolinium enhanced (LGE)

## Abstract

Hypertrophic cardiomyopathy is the most common genetic cardiac disorder and is defined by the presence of left ventricular (LV) hypertrophy in the absence of a condition capable of producing such a magnitude of hypertrophy. Over the past decade, guidelines on the screening, diagnostic, and management protocols of pediatric primary (i.e., sarcomeric) HCM have undergone significant revisions. Important revisions include changes to the appropriate screening age, the role of cardiac MRI (CMR) in HCM diagnosis, and the introduction of individualized pediatric SCD risk assessment models like HCM Risk-kids and PRIMaCY. This review explores open uncertainties in pediatric HCM that merit further attention, such as the divergent American and European recommendations on CMR use in HCM screening and diagnosis, the need for incorporating key genetic and imaging parameters into HCM-Risk Kids and PRIMaCY, the best method of quantifying myocardial fibrosis and its prognostic utility in SCD prediction for pediatric HCM, devising appropriate genotype- and phenotype-based exercise recommendations, and use of heart failure medications that can reverse cardiac remodeling in pediatric HCM.

## 1 Introduction

Hypertrophic cardiomyopathy (HCM) is the most common genetic heart disease, with a prevalence of 0.2% and an incidence of approximately 1 per 500 adults (1). These figures are likely underestimated due to the large number of asymptomatic cases; the actual prevalence of the disease is much higher when genetic testing and contemporary imaging techniques are applied (1). The pathogenesis of adult HCM is linked to autosomal dominant mutations in various genes that encode sarcomeric proteins, most frequently beta myosin heavy chain 7 (MHY7) and myosin-binding protein C3 (MYBPC3) (2). The clinical course of HCM is highly variable, ranging from asymptomatic to life-threatening complications such as sudden cardiac death (SCD), left ventricular outflow tract obstruction (LVOTO), heart failure with reduced ejection fraction (HFrEF), and atrial fibrillation with associated embolic events (3).

Compared to adults, pediatric HCM is an evolving field due to relatively limited data on epidemiology, etiologies, screening, diagnostic protocols, and management strategies. Epidemiological data from the United States indicate that HCM is substantially less common in children than adults, with an estimated prevalence of 1.2/1,000,000 and an annual incidence rate of 1.3/100,000 (4–6). Furthermore, the penetrance of child relatives of HCM patients is low during childhood and adolescence (7).

Pediatric HCM can be classified based on etiology into primary and secondary (8). Primary HCM can be due to sarcomeric mutations or can be idiopathic, which may be due to currently unidentified sarcomeric mutations or non-sarcomeric variants (9). Primary HCM is sometimes called familial HCM because of its typical autosomal dominant pattern of inheritance. The clinical outcomes of familial HCM due to sarcomeric mutations or idiopathic HCM are very similar, hence the two are often grouped as primary familial HCM (8). Primary HCM is typically asymptomatic through the first years of life, appearing during late childhood or adolescence, and exhibits an annual mortality rate of ∼1%, which is not significantly different from adult HCM. On the other hand, secondary HCM due to glycogen storage diseases, lysosomal storage diseases, syndromic cases, fatty acid oxidation disorders, and endocrine disorders like acromegaly typically manifests during infancy and is associated with a high mortality rate (10–12).

This review focuses on primary familial HCM, either with an identified pathogenic/likely pathogenic (P/LP) sarcomeric genetic variant or idiopathic. We survey the existing literature on the screening and diagnostic approaches for childhood-onset HCM and SCD risk assessment in such cases and highlight open questions in these areas.

## 2 HCM diagnosis and risk assessment

### 2.1 Cardiac MRI in HCM diagnosis

The diagnostic criterion for HCM in children is different than in adults. Left ventricular (LV) thickness children is adjusted for body size and growth, with a thickness >2 standard deviations (SD) above the mean after adjustment considered diagnostic for HCM (13–15), Some flexibility can be employed in the SD criterion when there is a high pretest probability of HCM like a strong family history or a positive cascade genetic test (16). Transthoracic echocardiography (TTE) is the primary imaging modality for diagnosing HCM but has limitations like poor acoustic windows and an overestimation of wall thickness on oblique sections (17–24).

Cardiac MRI (CMR) better distinguishes between the epicardial and endocardial layers to offer detailed information about LV systolic and diastolic function, LVOTO severity, atrial enlargement, and mitral regurgitation (17). CMR is also effective in detecting LV aneurysms, mural thrombi, and papillary muscle abnormalities in patients with sarcomeric HCM (13, 16). Existing data in adults with suspected HCM have showed that CMR leads to a clear HCM diagnosis in 44.7% of patients, indicated an alternative diagnosis in 5.3% of patients, and demonstrated no significant hypertrophy was found in 20.4% of patients, thereby refuting TTE findings (25). It will be important to determine whether these findings translate to pediatric HCM patients. Nevertheless, recommendations on when CMR is indicated for diagnosis vary. The 2014 and 2023 ESC guidelines recommend conducting CMR all HCM patients at initial evaluation to establish a baseline (13, 15), while the 2020 AHA/ACC recommends reserving CMR for cases where TTE findings are inconclusive (16). The 2023 ESC guidelines recommend considering CMR every 2–5 years to monitor disease progression on a case-by-case basis (class IIa recommendation) (15).

Given that phenotypic manifestations of HCM in children are more subtle than in adults, the question arises whether all children with suspected or diagnosed HCM should undergo CMR, as many of the aforementioned manifestations may be missed by TTE. Hence, studies evaluating the diagnostic yield and prognostic value of CMR, especially when TTE is negative or inconclusive, are needed in pediatric HCM patients. CMR can visualize subtle morphological changes in HCM genotype-positive individuals who do not have LV hypertrophy, a situation that often arises in children. These changes include narrow blood-filled myocardial crypts (i.e., deep, blood-filled invaginations within the LV myocardium), elongated mitral leaflets, and expanded extracellular space (23, 26–28). Lorenzini et al. showed that 32% of sarcomeric mutation carriers (median age 14.2 years) who were HCM phenotype-negative at first evaluation on TTE fulfilled diagnostic criteria of HCM on CMR (29). Furthermore, younger age at HCM diagnosis and sarcomeric mutations are predictive of long-term adverse outcomes including heart failure, atrial fibrillation, and ventricular arrhythmias (30). These findings strongly support the ESC guidelines that CMR be conducted in all genotype-positive children (15). However, there is currently insufficient evidence on the benefit of CMR in aiding the diagnosis of familial HCM without a genetic diagnosis (class IIb recommendation) (15).

**Figure 1:**
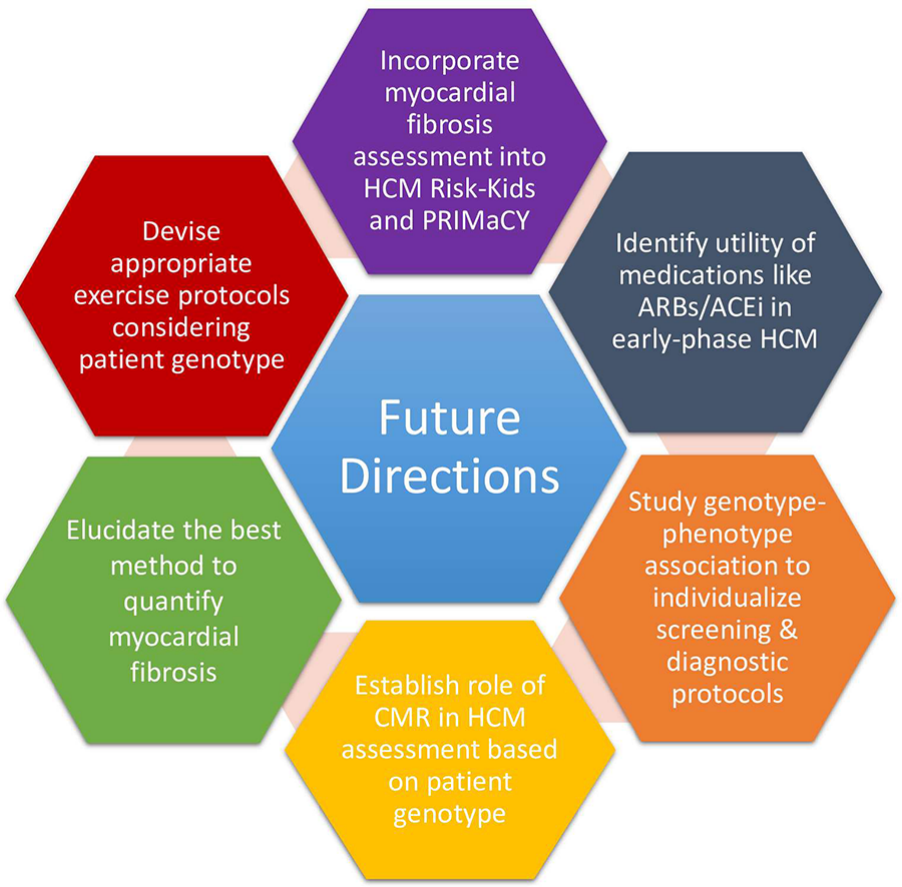
Proposed future directions in the field of pediatric HCM.

Different HCM genotypes can differentially affect cardiac anatomy and physiology, manifesting as different degrees of myocardial perfusion alterations, fibrosis, and diastolic dysfunction. Increasing consideration of these changes as part of the HCM spectrum may result in a broadening of what defines HCM beyond simply LV hypertrophy (9). We posit that CMR would become key in this context by providing a more comprehensive description of the cardiac anatomy (Figure 1). CMR itself is a tool to explore the genotype-phenotype association, exemplified by the use of different CMR techniques to associate differet genotypes (e.g., sarcomeric vs. non-sarcomeric) with divergent findings on myocardial oxygenation (31). The translational potential of genotype-phenotype studies is illustrated by the results of the randomized, double-blinded, placebo-controlled trial showing that mavacampten—a first-in-class drug targeting cardiac myosin ATPase tailored to the pathophysiology of sarcomeric HCM—significantly improved exercise capacity, LVOTO, NYHA functional class, and health status in patients with hypertrophic obstructive cardiomyopathy (HOCM) (32).

### 2.2 Sudden cardiac death risk assessment

Risk factors for SCD vary between adults and pediatrics, which are reflected in different recommendations on SCD risk assessment in both groups. Approaches for SCD risk assessment in adults include the six-parameter risk score recommended by the 2011 AHA/ACC guidelines, the HCM Risk-SCD score recommended by the 2014 and 2023 ESC guidelines, and the enhanced 2020 AHA/ACC SCD risk assessment approach (14, 33, 34). For children, the 2020 AHA/ACC guidelines recommend ICD placement with HCM children who have ≥1 major risk factor for SCD, including a family history of SCD, massive LVH ≥30 mm in any LV segment, syncope, LV apical aneurysm, and LV systolic dysfunction. However, these risk factors were largely derived from studies conducted on adult patients (16). The phenotypic characteristics of HCM in children differ from those in adults (see above), hence the applicability of adult risk factors in pediatric cases may be limited (35–37). The need for devising SCD assessment approaches tailored to pediatric HCM is underscored by the higher risk of SCD in children (0.8%–2%) than in adults (<0.8%) (38, 39). Furthermore, children are ∼36% more likely to experience arrhythmic events compared to adults (38, 39). Children ICD recipients face a lifetime of device-related complications, including lead fracture/failure, infective endocarditis, or the need for lead repositioning, for which there are currently no preventative approaches (39).

Unique risk factors for SCD in the pediatric population include LV posterior wall diameter, left atrial diameter, and LVOTO, which are not risk factors for SCD in adult HCM (40). Conversely, family history of SCD and abnormal blood pressure response to exercise are not significant risk factors for SCD in HCM children (41). The evidence behind age as a risk factor for SCD in children is weak, hence it is not incorporated in current risk prediction models (discussed below) (42). A meta-analysis by Norrish et al. reported that SCD risk factors with sufficient evidence for use in pediatric HCM patients include previous ventricular tachycardia/ventricular fibrillation, unexplained syncope, non-sustained ventricular tachycardia, and extreme LVH (37). Subsequent studies identified left ventricular posterior wall diameter, left ventricular outflow tract gradient, and, myocardial fibrosis as additional risk factors (40, 43). Furthermore, although the meta-analysis by Norrish et al. did not find an abnormal blood pressure response to exercise to be a significant predictor of SCD (37), a recent study evaluating a cohort of 630 primary HCM pediatric HCM patients <18 years demonstrated that abnormal exercise stress test results were present in ∼28% of patients, with exercise stress test-induced ischemia being independently associated with lower SCD-free survival (HR, 3.32; 95% CI, 1.27–8.70) (44). It is important to note that the meta-analysis by Norrish et al. was based on a limited number of studies which were limited in terms of their patient selection, sample size, and follow-up times. Therefore, our understanding of the risk factors for SCD in pediatric HCM patients continues to evolve through robustly designed multi-center studies.

Two models exist that enable individualized SCD risk assessment in pediatric HCM (Table 1). Norrish et al. developed a risk prediction model for SCD in children called HCM Risk-Kids. This model achieved a c-statistic—which is a measure of the discriminative ability of a risk prediction model—of 0.69, with 1 patient being saved for every 10 ICD implantations in patients with a ≥6% 5-year risk of SCD (35). HCM Risk-Kids has been externally validated on a cohort of 421 HCM patients aged 1–16, with the 5-year SCD risk cut-off of ≥6% identifying 73.9% of SCD events with a c-statistic of 0.70 (45). Miron et al. developed another individualized SCD prediction model, PRIMaCY, which achieved a c-statistic of ∼70% in predicting 5-year SCD risk (40). No study has directly compared the performance of HCM-Risk kids and PRIMaCY in predicting the risk of SCD. These models were recently incorporated into the 2023 ESC cardiomyopathy guidelines, which recommended the use of either of these two models for HCM patients <16 years old (15).

**Table 1:** Comparison of the individualized SCI prediction models for pediatric HCM.

Characteristics	HCM-risk kids	PRIMaCY
Age range for use	≥1 years and ≤16 years	≤18 years
Predictor variables used **(Differences bolded)**	1. Unexplained syncope. 2. **Maximal left-ventricular wall thickness.** 3. Left atrial diameter. 4. Left ventricular outflow tract gradient. 5. Non-sustained ventricular tachycardia	1. **Age at diagnosis.** 2. **Interventricular septal thickness.** 3. **Left ventricular posterior wall thickness.** 4. Left atrial diameter. 5. Left-ventricular outflow tract gradient. 6. Non-sustained ventricular tachycardia 7. Unexplained syncope
Internal validation c-statistic	0.69 (95% CI = 0.66–0.72	0.75 (CI not provided)
External validation c-statistic	0.714 (95% 0.58–0.85)	0.71 (CI not provided)
Model website	https://hcmriskkids.org/	https://primacycalculator.com/
Future directions	• Incorporate CMR-based assessments of cardiac structure and function • Incorporate measures of myocardial fibrosis • Evaluate if incorporating EKG findings improve SCD prediction by these models • Update models according to future data on adverse outcome risk based on genetic basis of HCM	

Although these models have transformed pediatric HCM clinical practice, some notable limitations must be contended with (46). Most glaringly, important predictors of future adverse outcomes like electrocardiography parameters, CMR-based features, measures of myocardial fibrosis, and genetic factors like the presence of sarcomeric mutations have not been included (Figure 1). The importance of myocardial fibrosis and genetic factors is discussed below. Another critique raised by Maron et al. is concern over the derivation of HCM Risk-Kids from the HCM Risk-SCD score, which the authors found to lack sensitivity (47). However, the HCM Risk-SCD score has been externally validated in other multi-institutional studies and meta-analyses (48, 49). Although it is no longer the case that pediatric risk factors for SCD need to be derived from adult studies, the criteria by which variables were selected for the HCM Risk-Kids model was that they needed to be examined in at least two or more studies employing univariate or multivariate analyses. Employing only predictors established in multivariate analyses, although more robust, was not possible because of the limited data on pediatric HCM.

These findings highlight the transformative impact of individualized pediatric SCD assessment models but also raise suggestions on how to improve them. Future studies expanding our knowledge of the genotype-phenotype association in HCM may reveal important caveats about the genetic basis of SCD risk that need to be reflected in these models.

### 2.3 Myocardial fibrosis in SCD risk assessment

Myocardial fibrosis can be an indicator of myocardial ischemia, LV diastolic dysfunction, and future risk of atrial fibrillation and SCD (50). Late-gadolinium enhancement (LGE) is the most widely used tool to quantitatively measure myocardial fibrosis. Studies have demonstrated that LGE improves the stratification of adult HCM patients at low-to-intermediate risk of SCD when added to the 2011 AHA/ACC algorithm and HCM-Risk SCD (16, 51, 52). Consequently, the enhanced AHA/ACC SCD risk assessment approach incorporates LGE and LV apical aneurysm detected by CMR to the 2011 AHA/ACC six-parameter prediction score and is currently the most sensitive SCD risk assessment method for adult HCM patients (∼95%) (16, 34). Integrating artificial intelligence (AI) into CMR-LGE, a multi-center study on 1,229 HCM patients (mean age 52 years) showed that radiomic features—i.e., using computational algorithms to extract quantitative features from medical images—of myocardial scars on LGE-CMR added incremental prognostic value to HCM Risk-SCD and AHA/ACC SCD risk assessment protocols for adults (53). The 2023 ESC guidelines maintained the recommendation of utilizing HCM Risk-SCD as the first-line tool in SCD risk assessment in adult HCM patients, but state that the presence of extensive LGE (≥15%) in patients classified as low-risk can inform decision-making on prophylactic ICD implantation (15).

HCM Risk-Kids and PRIMaCY are yet to incorporate LGE into their risk prediction (26, 33, 34, 54). Low levels of myocardial fibrosis with LGE (>2% of LV mass) are common in children with HCM and the introduction of LGE significantly improves the predictive accuracy of the HCM Risk-Kids prediction model (55, 56). A recent study on a cohort of 166 pediatric HCM patients with a mean age of 10.4 years demonstrated the prognostic value of LGE in determining major cardiac events (i.e., sustained VT, resuscitated cardiac arrest, SCD, end-stage heart failure, heart transplant, and appropriate ICD intervention) (57). This study showed that the optimal cutoff LGE extent for predicting events was ≥2% (57). The predictive accuracy—evaluated by the median time-dependent area under the curve (AUC)—of LGE extent (0.88, 95% CI 0.86–0.89) significantly outperformed that of syncope (0.63, 95% CI 0.61–0.66, *p* < 0.0001) and nonsustained ventricular tachycardia (0.52, 95% CI 0.50–0.53, *p* < 0.0001), both of which have been included in the individualized pediatric SCD predictive models (57). Although this study utilized a composite primary endpoint not composed of just SCD or its equivalent event (i.e., sustained VT, resuscitated cardiac arrest, or appropriate ICD intervention) but also end-stage heart failure and heart transplantation, these results advocate for the introduction of LGE in the SCD risk assessment for pediatric HCM (Figure 1).

The influence of genetic background such as the presence of sarcomeric mutations on the decision to perform LGE is an area of active interest. LGE may be less prevalent in pediatric HCM (46%) compared to adults (∼60%), particularly in sarcomeric mutation carriers without overt left ventricular hypertrophy (58). However, some pediatric patients demonstrate progression of LGE extent over time, although age at diagnosis or time elapsed since diagnosis was not predictive of LGE increase, indicating a potential genetic basis for the progression of myocardial fibrosis in HCM (58). These results highlight the need for further exploration of genotype-phenotype associations with respect to myocardial fibrosis development and progression, which may enhance the decision-making process of when to opt for LGE based on patient genotype.

There is no universally accepted/agreed-upon method of quantifying myocardial fibrosis. LGE may not be the most sensitive method for detecting fibrosis. Alternative approaches like T1 mapping or calculation of extracellular volume (ECV) fraction may be more sensitive for assessing diffuse interstitial fibrosis (59). For example, left atrial enlargement and diastolic dysfunction can be present in childhood-onset HCM without LVH, which may be due to diffuse interstitial fibrosis that is undetectable by LGE (58, 60). In adult HCM patients, T1 mapping and ECV fraction measurements have been associated with major cardiac events like SCD in patients without LGE, even in those determined low-risk by the enhanced AHA/ACC strategy and HCM Risk-SCD (61, 62). Studies exploring what the added prognostic value of T1 mapping over LGE is in predicting SCD risk and other adverse cardiac events in pediatric primary HCM are needed (Figure 1). These alternate approaches may also constitute avenues to explore the genotype-phenotype association of HCM, as one study applied radiomics analysis to T1 mapping to study the genotype-phenotype associations of sarcomeric (MYH8 and MYPBC3) HCM patients with respect to myocardial fibrosis (63).

## 3 Appropriate screening age for children

Genetic evaluation of relatives of HCM patients requires a systematic approach including a comprehensive family history to assess for early-onset HCM and family history of SCD, a comprehensive phenotypic (i.e., clinical) and genetic evaluation of the proband to confirm phenotype-positivity and guide cascade genetic testing, referral for genetic counseling, and genotype- and phenotype-directed guidance on potential therapies like ICD, medications, and lifestyle modifications (64).

The 2011 AHA/ACC guidelines recommended screening first-degree child relatives with a positive HCM family history at the age of 12 years, with earlier screening recommended in children with a family history of SCD, participation in intense physical activities, and an early growth spurt (14). The 2014 ESC guidelines recommended initiating screening in first-degree child relatives with an unknown genetic history after the age of 10 years with screening intervals of 1–2 years between the ages of 10–20 and every 2–5 years thereafter (13).

Studies in the years following these guidelines challenged the notion that sarcomeric mutations and clinically significant adverse events in children with familial HCM are rare. Approximately 43%–63% of pediatric HCM cases are associated with sarcomeric mutations, and sarcomeric childhood-onset HCM is linked increased risk of heart failure and a composite clinical outcome of life-threatening ventricular arrhythmias, atrial fibrillation, stroke, and death (38, 65, 66). Childhood-onset HCM also exhibits a steeper increase in LV wall thickness and higher median event rate than adults, associated with a greater likelihood of developing life-threatening ventricular arrhythmias and interventions such as heart transplantation or ventricular assist device implantation (38, 67). Furthermore, a significant proportion of HCM phenotype-positive children and those with major adverse cardiac events were below the recommended screening age of 10 years, with around 69% of these children meeting the 2011 AHA/ACC and 2014 ESC criteria for early screening (67, 68).

Considering these findings, the updated 2020 AHA/ACC guidelines recommended clinical and/or genetic screening first-degree children relatives of genotype-positive patients irrespective of age. All children and adolescents with a family history of early-onset HCM are also advised to undergo screening regardless of age (16). Echoing the trend of basing screening decisions off proband genetic testing results rather than a cut-off age, the 2023 ESC cardiomyopathy guidelines recommended that if a P/LP genetic variant is identified, cascade genetic testing should be performed in first-degree relatives irrespective of age. Genotype-positive relatives should undergo clinical evaluation by EKG, multimodality imaging (echocardiography and CMR), and long-term follow-up, while relatives without the disease-causing variant are discharged from further follow-up but counseled to seek re-assessment if they develop symptoms (15). The value of genetic testing at a young age is further indicated by a study showing that a younger age at HCM diagnosis and sarcomeric mutations are predictive of long-term adverse outcomes including heart failure, atrial fibrillation, and ventricular arrhythmias (30). In favor of discharging genotype-negative patients, Nielsen et al. showed that first-degree relatives of an index HCM patient with no P/LP variant have a low frequency of diagnosis at initial evaluation and risk of developing the condition during 5 years of follow-up, and, if diagnosed, are at low risk of SCD (69). On the other hand, even sarcomeric variants of undetermined significance (VUS) have been shown to correlate with adverse outcomes (30), hence child relatives of the index VUS HCM patient should be offered serial clinical evaluations because of age-related penetrance (15). Finally, if no P/LP variant is identified in the proband or if genetic testing is not performed, then clinical evaluation with EKG and multi-modality imaging should be performed in first-degree relatives (class I recommendation) (15).

A puzzling HCM patient population that requires further improvement in their management is genotype-positive phenotype-negative patients. More precision is needed when ascertaining their risk of phenotypic conversion, which is affected by the specific underlying genetic mutation and possibly by gender. A retrospective study following 285 phenotype-negative sarcomeric mutation carriers demonstrated that 46% of individuals develop HCM over 15 years of follow-up (29). Male sex and an abnormal EKG were independently associated with higher penetrance. Furthermore, the *TNNI3* sarcomeric protein variants had the lowest penetrance (29). Hence, future updates must account for individual differences in risk of phenotypic conversion, perhaps reflected as a stronger emphasis on the use of CMR and myocardial fibrosis assessment in high-risk groups (Figure 1).

Early genetic screening of children is not without its challenges (Table 2). It may not be feasible to screen every first-degree child relative of the index HCM patient. Many families also choose not to undergo genetic testing, limiting the identification of P/LP variants and cascade genetic testing in relatives (70). Clinical and imaging evaluation of children should take place in these circumstances. The financial and psychological cost of universal screening need also be considered, especially because significant LV hypertrophy and adverse events like SCD—although more common than previously thought—are still exceedingly rare before 10 years of age (70). The psychological aspects of an HCM diagnosis are increasingly recognized, such as anxiety among the child and especially parents, a difficult transition to higher levels of education and from pediatric to adult care, and the social stigma associated with cardiovascular disease and its consequences on confidence and anxiety, especially around exercise. Support from a trained professional like a clinical psychologist has been shown to significantly mitigate quality of life impairment in children who receive an HCM diagnosis (71, 72). On the other hand, genetic and clinical screening to identify potentially affected child relatives of the affected HCM individual does not seem to impair quality of life (72). Lastly, universal early screening could promote the potentially unnecessary prescription of medical treatment, lifestyle changes particularly surrounding exercise, and prophylactic implantation of ICDs. A paradigm shift towards individualized risk assessment and shared decision-making is emphasized because indiscriminate screening in an era where judicious resource allocation is crucial does not seem feasible.

**Table 2:** Potential benefits and harms of early screening for HCM in children.

Benefits	Harms
Decrease in uncertainty regarding HCM status	Increased anxiety about the child's future
Anticipating future care enhances patient management through informed decision-making	Alteration of one's self-image and stigma associated with cardiovascular disease
Early detection and management with possible improved prognosis	Lack of confidence around exercise/physical activity can negatively impact quality of life
Overall reduced healthcare costs by reducing the need for advanced, lifelong therapies for complications	Increased insurance costs and accessibility limitations

To address many of these challenges, we need to understand the link between specific genotypic variants, phenotypic HCM manifestations, and associated risks of adverse events (9). Genetic studies on the penetrance of different P/LP variants, most of which are currently unknown (15), are required to inform screening decisions (Figure 1). Further research may expand the definition of HCM beyond LV hypertrophy since different genotypes can differentially affect various aspects of cardiac structure and function and thus may be detected differently during screening (9).

## 4 Management of pediatric HCM

### 4.1 Exercise recommendations

HCM was originally described in the context of SCD and was popularized as the most common cause of SCD in professional athletes (73). Also, exercise can theoretically trigger hypertrophy of the myocardium, which may exacerbate LVOTO. Consequently, management strategies have historically strictly advised against any form of exercise other than low-intensity training, stemming from the fear of triggering a ventricular arrhythmia and SCD (13, 14). Subsequent studies began to highlight the negative psychological and long-term medical outcomes HCM patients may be predisposed to because of this restriction on their lives. Furthermore, a substantive percentage of HCM patients nowadays are largely asymptomatic and exhibit a normal life expectancy. It thus becomes important to explore more deeply the types of physical activity that HCM patients can carry out, especially during childhood and adolescence given its enormous benefits on physical and social development in this phase.

Recent studies have begun to show that exercise training during childhood and adolescence is associated with favorable indices of diastolic function independent of LVH (74). This is consistent with data on adult HCM patients, where a sedentary lifestyle is associated with obesity and adverse cardiovascular outcomes on the one hand and exercise is correlated with improved exercise capacity and cardiovascular and quality-of-life outcomes on the other (75–83). Recent studies also indicate that even vigorous exercise in genotype-positive HCM patients across most age groups including children and adolescents does not increase the risk of a primary endpoint of death, resuscitated SCD, arrhythmic syncope, and appropriate shock from an ICD (76). Furthermore, endurance exercise in athletes with HCM is associated with an enlarged LV cavity size and amelioration of outflow obstruction (84). Consequently, the recent North American and European guidelines adopt somewhat of a more liberal approach and encourage low-to-moderate intensity for all HCM genotype-positive individuals, even those exhibiting the phenotype (15, 16). Competitive and high-intensity sport is still approached with caution and is allowed for phenotype-negative and low-risk pediatric and adult phenotype-positive individuals after extensive initial assessment and re-assessments to check for phenotypic progression (15). For future studies, in line with the emphasis on genotype-phenotype associations and tailored management strategies, it will be important to distinguish exercise recommendations for different genotypes and for pediatric patients in different risk categories of the individualized SCD risk models.

### 4.2 Medications

Therapy for heart failure does not distinguish between specific etiologies and can be grouped into medical therapy, resynchronization therapy, ventricle assist devices, and transplantation. Different medications indicated for the treatment of HFrEF include angiotensin-converting enzyme inhibitors (ACEi), angiotensin receptor blockers (ARBs), angiotensin receptor neprilysin inhibitors (ARNI), beta blockers, mineralocorticoid receptor antagonists, and sodium-glucose cotransporter-2 (SGLT2) inhibitors (85, 86). These medications are also indicated in end-stage HCM with LV systolic dysfunction (86).

The role of these medications in early-stage HCM, which often is the case in pediatric patients, is not well understood. The multicenter, randomized, double blind, placebo controlled, phase 2 VANISH clinical trial of 178 participants including both adults and children with early-stage HCM showed that valsartan treatment for 2 years improved cardiac structure and function, indicated by an integrated *z*-score of LV wall thickness, mass, and volume, left atrial volume, doppler systolic and diastolic velocities, and serum levels of hs-troponin T and pro-BNP (87). In contrast, the INHERIT clinical trial did not show a statistically significant benefit to losartan use in decreasing LV mass in middle-aged patients with overt HCM compared to placebo (88). The INHERIT trial utilized CMR and cardiac CT to measure the primary outcome of LV mass, while secondary outcomes included LV fibrosis, maximum LV wall thickness, left atrial volume, and plasma levels of NT-pro-BNP. Therefore, different outcome measurements may have contributed to the conflicting results of the VANISH and INHERIT studies. However, a recent analysis showed that utilizing the VANISH composite *z*-score in the INHERIT cohort still did not result in a statistical benefit to losartan use (89).

It is plausible that ACEi/ARB may be beneficial in reversing cardiac remodeling in early-phase disease but this effect is lost upon progression to overt HCM (90). However, the recent multicenter, double-blind, placebo-controlled VANISH randomized clinical trial of 34 sarcomeric HCM patients (mean age of 16 years) showed no statistical benefit to valsartan use in patients with early subclinical HCM with no LVH using the aforementioned integrated *z*-score approach (91). However, this study was underpowered to detect a statistically significant benefit due to a small sample size, short follow-up duration (∼2 years), and slow phenotypic progression in both the valsartan and placebo group. Larger scale studies with longer follow-up durations are required to conclusively assess the clinical benefit of valsartan on cardiac remodeling in early phase HCM. It will also be important to determine if the underlying genotype influences treatment response in the early phase.

## 5 Conclusions

Tremendous advancements have been made in the screening protocols, timely diagnosis, and management of pediatric HCM, a historically understudied field. The recent ESC cardiomyopathy guideline update embraced the individualized pediatric SCD prediction models, but the incorporation of key imaging and genetic parameters into these models is awaited. Other important knowledge gaps notably include gaining a better understanding of the genotype-phenotype association, the best technique to detect myocardial fibrosis, and defining the potential role of AI as clinical decision support systems in screening and as algorithms like LGE-CMR radiomics approaches. Addressing these challenges will undoubtedly contribute to improving the care of these patients and thereby alleviate a significant healthcare burden in terms of morbidity, mortality, and health expenditures.
